# Fibrosis after Myocardial Infarction: An Overview on Cellular Processes, Molecular Pathways, Clinical Evaluation and Prognostic Value

**DOI:** 10.3390/medsci9010016

**Published:** 2021-03-01

**Authors:** Renato Francesco Maria Scalise, Rosalba De Sarro, Alessandro Caracciolo, Rita Lauro, Francesco Squadrito, Scipione Carerj, Alessandra Bitto, Antonio Micari, Gianluca Di Bella, Francesco Costa, Natasha Irrera

**Affiliations:** 1 Department of Clinical and Experimental Medicine, Policlinic “G. Martino”, University of Messina, 98100 Messina, Italy; rfm.scalise@gmail.com (R.F.M.S.); rosalba.desarro@icloud.com (R.D.S.); caracciolo.alessandro.ac@gmail.com (A.C.); scarerj@unime.it (S.C.); gianluca.dibella@tiscali.it (G.D.B.); nirrera@unime.it (N.I.); 2 Section of Pharmacology, Department of Clinical and Experimental Medicine, University of Messina, 98100 Messina, Italy; lrritalauro@gmail.com (R.L.); fsquadrito@unime.it (F.S.); abitto@unime.it (A.B.); 3 Department of Biomedical and Dental Sciences and Morphological and Functional Imaging, University of Messina, A.O.U. Policlinico “G. Martino”, 98100 Messina, Italy; micariantonio@gmail.com

**Keywords:** myocardial infarction, inflammation, fibrosis, molecular pathways, cardiac magnetic resonance, prognostic value

## Abstract

The ischemic injury caused by myocardial infarction activates a complex healing process wherein a powerful inflammatory response and a reparative phase follow and balance each other. An intricate network of mediators finely orchestrate a large variety of cellular subtypes throughout molecular signaling pathways that determine the intensity and duration of each phase. At the end of this process, the necrotic tissue is replaced with a fibrotic scar whose quality strictly depends on the delicate balance resulting from the interaction between multiple actors involved in fibrogenesis. An inflammatory or reparative dysregulation, both in term of excess and deficiency, may cause ventricular dysfunction and life-threatening arrhythmias that heavily affect clinical outcome. This review discusses cellular process and molecular signaling pathways that determine fibrosis and the imaging technique that can characterize the clinical impact of this process in-vivo.

## 1. Introduction

**Figure 1:**
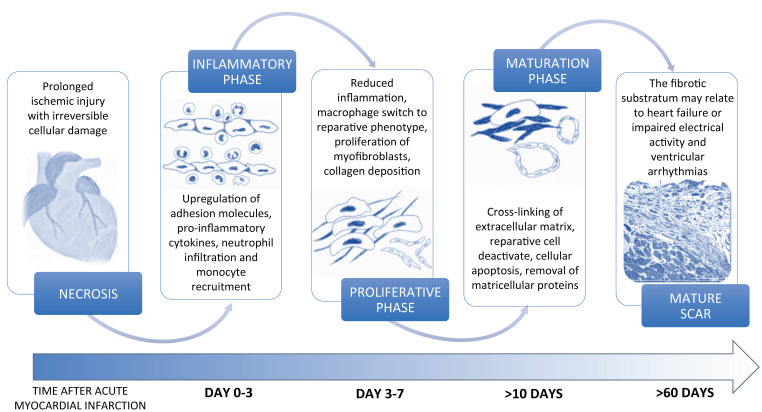
Different phases of the healing process after myocardial infarction.

Myocardial infarction (MI) is a leading cause of mortality and morbidity in industrialized countries and represents the first cause of heart failure (HF) worldwide [1]. The myocardial ischemic injury causes cardiomyocyte (CM) necrosis, which triggers molecular signals of damage and the consequent activation of an intense inflammatory response aimed at tissue repair. The myocardium has a poor regenerative capacity in adult mammalian tissue because of the post-mitotic nature of CM. Consequently, a fibrotic scar replaces the lost cells after CM necrosis [2]. At the beginning of the wound healing process, immune cells are activated and recruited by damage signals in the necrotic area in order to remove dead cells and interstitial debris through phagocytosis and proteolysis (Figure 1).

Subsequently, anti-inflammatory immune cells secrete cytokines that attract and activate reparative cells to produce extracellular matrix (ECM) [3]. Lastly, the apoptotic process is activated, and the fibrotic scar maturation begins. The balance between the inflammatory and reparative phases regulates the healing process and has a significant impact on the quality of the scar. Fibrosis represents one of the main mechanisms involved in the repairing process following MI and may appear as replacement and/or reactive fibrosis [4]. In particular, replacement fibrosis is responsible for the substitution of cardiomyocytes in the necrotic area with a fibrotic scar formation, which prevents ventricular wall rupture and adverse remodeling. Reactive fibrosis causes interstitial fibrosis both in the infarct border zone and in the surrounding myocardium as a consequence of an incomplete inflammation resolution or excessive reparative process [5]. Both replacement and reactive fibrosis may affect ventricular structure and systo-diastolic function, thus providing the physiopathological substratum of ischemic HF [6,7]. Furthermore, fibrotic scar and interstitial fibrosis may affect electrical myocardial activity and predispose to life-threatening ventricular arrhythmias [8]. In this review, we will focus on the cellular processes and the molecular signaling pathways leading to fibrosis after MI and will provide a clinical outlook on the diagnostic techniques and the prognostic impact of fibrosis based on current imaging techniques.

## 2. Cellular Processes Leading from MI to Fibrosis

### 2.1. Inflammatory Phase

The healing process consequent to MI is regulated by several pathophysiological factors. In the very early phase after MI, necrotic cells and injured ECM release damage signals, identified as Danger Associated Molecular Patterns (DAMPs) which are recognized by innate immune system cells through specific Pattern Recognition Receptors (PRRs), with the consequent activation and amplification of the inflammatory response [9]. Dendritic cells and macrophages, which primarily recognize and internalize DAMPs, initiate the recruitment of other inflammatory cells presenting these antigens [10]; dendritic cells concentration correlates with the number of different cell types activated following MI, such as macrophages, neutrophils, and T-cells in the necrotic and fibrotic areas, also on the basis of the extent of damage [11,12,13]. The complex inflammatory mechanism is related to DAMPS release and inflammatory cells stimulation but also to the simultaneous upregulation and secretion of pro-inflammatory cytokines including chemokines, adhesion molecules, and interleukins: all these molecules drive and amplify the inflammatory response recruiting and activating leukocytes in the infarcted area [14]. Chemokines represent a strong chemoattractant for leucocytes, particularly for neutrophils and monocytes. Neutrophils provide for clearance of cellular and ECM debris from the wound and are also responsible for the release of proteolytic enzymes. In particular, neutrophil matrix metallopeptidases 2 and 9 metabolize ECM components promoting the removal of dead cells and inhibit neutrophil apoptosis facilitating the self-maintenance of the inflammatory response. However, the overexpression of neutrophil metallopeptidases has been related to excessive ECM degradation and adverse cardiac remodeling [15]. Over and above that, neutrophils play a key role in the inflammation shutdown and simultaneous initiation of the reparative phase. In fact, neutrophils may stimulate the anti-inflammatory and pro-fibrotic activity of macrophages, thus counteracting inflammation and stimulating the reparative process [16,17]. Both monocytes and macrophages are involved in the inflammatory response and in the reparative process with two main differentiation phenotypes: M1, with pro-inflammatory features, and M2, with anti-inflammatory and pro-fibrotic features [2,18]. In the acute phase, monocytes and M1 macrophages phagocyte dead cells, recruit other leukocytes in the necrotic area and produce proteases [19,20] that contribute to clear cellular and ECM debris preparing the ground for the granulation tissue [14]. 

### 2.2. Reparative Phase

Subsequently, the macrophage population shifts from M1 to M2 phenotype and secretes mediators that promote the reparative process, such as TGF (Transforming Growth Factor) β and IL (interleukin) 10 [2,19]. A paramount role in the reparative processes is attributed to TGFβ that induces fibroblasts differentiation into myofibroblasts and stimulates the release of type I collagen and connective tissue growth factor (CTGF); moreover, TGFβ decreases metalloproteinases levels with consequent collagen accumulation [21]. Therefore, the unbalanced activation of TGFβ may exacerbate fibrotic process worsening the quality of the scar; in addition, TGFβ over-activation might be considered as a negative predictor factor for heart failure. IL 10 raises M2 macrophages concentration, reduces proteinases secretion, and boosts fibroblasts migration and proliferation [22]. Moreover, IL10 qualitatively influences fibroblast activity through a reduction of the collagen I/collagen III ratio that enhances tissue compliance and myocardial function. Interestingly, it has been demonstrated that macrophages are able to shift into a fibroblast-like phenotype characterized by an upregulation of specific genes coding for ECM proteins and molecules involved in ECM synthesis control [18,23]. A direct correlation between macrophage concentration and collagen deposition has been demonstrated [18]. Also mast cells contribute to the healing process; their activity is related to the secretion of proteases and mediators such as histamine, tryptase, and chymase. Histamine inhibits TGFβ through STAT6 pathway, thus reducing fibrotic process stimulation [24], whereas tryptase and chymase may promote fibrosis through TGFβ activation [25,26]. In particular, chymase converts angiotensin I into angiotensin II that promotes collagen synthesis. The reparative and inflammatory processes are also regulated by different T-cells: Th1 and CD8+ cells are activated in the acute phase, whereas Th2 and Treg are mainly involved in the chronic phase [27,28]. Th1 cells produce interferon-γ that, in turn, inhibits TGFβ and modulates Th2 cell activity, limiting secretion of IL 4 and 13 as pro-fibrotic mediators [29,30]. The altered Th1 cells activity may unbalance ECM turnover and might cause cardiac rupture [31]. Conversely, interferon-γ promotes Treg population differentiation that reduces Th1 and CD8+ activity, thus promoting a harmonic healing process [32,33]. In fact, CD8+ T cells remove cellular debris and promote collagen maturation [34]. As mentioned, Th2 cell activation may contribute to IL 4 and 13 release as pro-fibrotic factors that highly stimulate collagen synthesis [29] and recruit macrophages stimulating their differentiation in M2 subtype [35,36]. Tregs may contribute to the fibrotic process through TGF-β stimulation and IL-10 inhibition [37,38]; moreover, it has been demonstrated that these cells may promote M2 macrophage polarization, thus contributing to cardiac fibrosis [39]. The complex mechanisms that regulate the inflammatory response and the shift to reparative phase are strictly regulated to obtain optimal healing. In this dynamic scenario, ECM influences cellular activity and phenotypes. Collagen, fibronectin and hyaluronan fragments produced during ECM degradation in the necrotic area stimulate chemokines and cytokines synthesis in the immune and endothelial cells [40]. As ECM fragments are removed, a transitional matrix, mainly composed of fibrin and fibronectin, is produced by reparative cells as a plastic scaffold that facilitates migration and proliferation of other cells involved in the reparative process [41]. In this phase, ECM is rich in matricellular protein that binds several cell receptors involved in inflammatory and fibrotic processes [42,43]. Matricellular protein and TGFβ recruit and stimulate fibroblasts to differentiate into myofibroblasts that are phenotypically characterized by the expression of stress fibre and contractile proteins [44,45]. 

### 2.3. Maturative Phase

Cell proliferation is followed by a maturation phase of the scar: ECM structures crosslink stabilizing the scar tissue, reparative cells deactivate and undergo cell death, and finally, matricellular proteins are removed. The altered balance of these phases may worsen the inflammatory response that, in turn, may contribute to the activation of a detrimental reactive fibrosis [46,47]. Disproportionate collagen deposition and over-contraction of fibrotic tissue may cause an excessive stiffness of the scar that in turn may affect cardiac compliance and diastolic and systolic function [48]. On the contrary, insufficient collagen deposition and cross-linking are related to the appearance of a vulnerable scar prone to dilation and wall rupture [33,49,50].

## 3. Molecular Signaling Pathways

**Figure 2:**
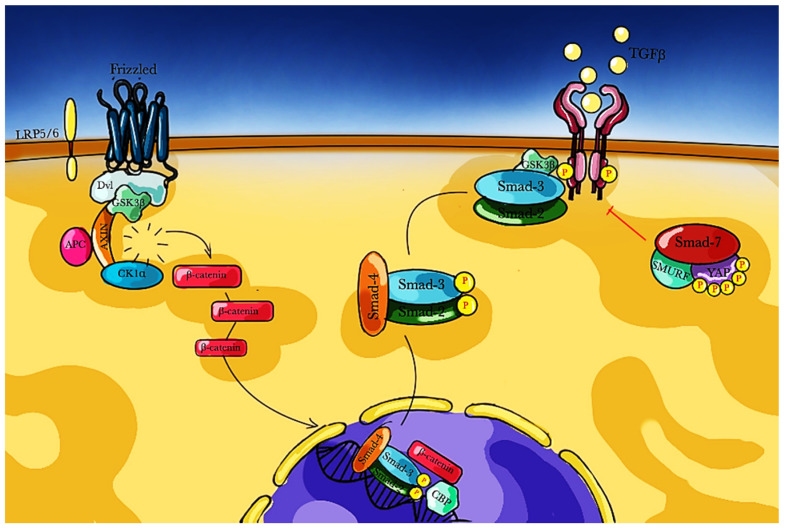
WNT/ β catenin pathway and its relation to gene transcription driving fibrogenesis. APC: Adenomatous polyposis coli protein; CK1α: Casein kinase 1α; DVL: Dishevelled protein; GSK3 β: Glycogen synthase kinase 3 β; LRP: Lipoprotein receptor-related proteins; P: phosphorus; SMAD: small mother against decapentaplegic protein; TGFβ: tumor growth factor β; YAP: yes-associated protein.

The key event that marks the beginning of the fibrotic process is the engagement of specific receptors by pro-fibrotic factors that activate downstream signals and regulate gene transcription (Figure 2). 

### 3.1. TGF-β Pathway

TGF-β is one of the most important mediators involved in fibrogenesis; 3 isoforms are expressed in mammalian, known as TGF-β1, TGF-β2, and TGF-β3, encoded by different genes [51,52]. TGF-β acts as a pleiotropic signal of different effects; the interaction with its specific receptor (i) may induce signals of migration/differentiation and may stimulate integrin expression, thus promoting adhesion process [53,54], (ii) regulates macrophages function stimulating the release of pro-fibrotic mediators [55,56], (iii) depresses T cell proliferation through IL-2 inhibition [57,58,59,60], (iv) promotes the shutdown of the inflammatory response by inhibiting pro-inflammatory cytokines and chemokines expression [61], (v) induces fibroblasts differentiation into myofibroblasts, thus inducing α-smooth muscle actin (α-SMA) and ECM gene transcription [62], (vi) influences cardiomyocytes inducing cardiac hypertrophy [63,64], (vii) reduces ECM turnover modulating the expression of tissue inhibitor of metalloproteinases (TIMPs) and plasminogen activator inhibitor (PAI)-1 [65]. The relevance of the overall effects of TGF-β on fibrosis is confirmed by different in vitro and in vivo experimental models. For instance, TGF-β1 deficient mice showed a reduced age-related myocardial fibrosis and higher cardiac compliance compared to wild-type mice [66], whereas mice with enhanced TGF-β1 expression showed a significant increase of fibroblasts concentration and, as a consequence, an increased myocardial fibrosis [67]. These experimental data indicate TGF-β1 involvement in fibrotic processes. TGF-β carries out its function by binding TGF-β type II receptor, constituting a stable and active complex that phosphorylates TGF-β type I receptor. TGF-β type I receptor is responsible for the activation of downstream cascade through its kinase activity that is directed to protein members of the Smad family [68]. Particularly, once activated, TGF-β type I receptor mediates Smad2 and Smad3 release that, following activation of Smad4, form a complex that subsequently moves into the nucleus and recruit transcription factors that modulate target gene expression. Other members of Smad family, such as Smad6 and Smad7, have an inhibitory activity towards the TGF-β type I receptor and interfere for transcription of Smad2 and Smad3. Moreover, Smad6 and Smad7 may induce degradation of activated TGF-β type I receptors [69]. Smad proteins finely regulates gene transcription involving co-activators and co-repressors that bind promoters and enhancers of specific DNA sequences, thus inducing collagen 3, junB and PAI-1 expression [70]. However, TGF-β signaling cascade can also be activated independently of Smad-pathway [71]. TGF-β type II receptor can activate TGF-β-activated kinase 1 (TAK1) that acts on MKK3/6 or MKK4 promoting the phosphorylation of the mitogen-activated protein kinases (MAPKs) JNK and p38 [72]; JNK and p38, in turn, activate the activating transcriptional factor 2 (ATF2) that is a Smad mediator for gene transcription [71]. TAK1 overexpression has been associated to enhanced myocardial interstitial fibrosis [73]. 

### 3.2. Angiotensin Pathway

Increased levels of Angiotensin II have been detected in fibrotic cardiac tissue. In fact, Angiotensin II may be considered as a key mediator of myocardial fibrosis. Also, its specific receptors AT1 and AT2 are overexpressed during fibrosis [71,74]. These receptors show opposites effects: AT1 activates pro-fibrotic downstream cascade while AT2 counteracts AT1 pro-fibrotic pathway [75,76]. Angiotensin II stimulates fibroblasts proliferation and adhesion, thus inducing TGF-β1, PAI-1, and ECM production and deposition [77,78,79]. Angiotensin II pro-fibrotic effects are mainly mediated by TGF-β. In fact, Angiotensin II was not able to induce fibrosis in TGF-β1-deficient mice [80]. 

### 3.3. Wnt/β-Catenin Pathway

In the last years, it has been demonstrated that the activation of the Wnt/β-catenin pathway represents a pro-fibrotic signal. The Wnt pathway physiologically modulates cell proliferation, differentiation, and migration through two different cascades: the canonical pathway that involves β-catenin, and the non-canonical pathway that is β-catenin-independent. When Wnt binds its specific receptor Frizzled (Fz) and the lipoprotein receptor-related protein 5/6 (LRP5/6) co-receptor, a complex stabilizes cytosolic β-catenin which migrates into the nucleus and interacts with transcription factors in order to regulate specific gene expression [81,82,83]. Frizzled-related proteins (FRP) may contribute to the regulation of fibrotic processes: fibroblast with loss of sFRP-1 showed increased levels of α-SMA and collagen accumulation [84]; sFRP2 acts as Wnt signaling inhibitor, in fact, it may reduce fibrosis and improve cardiac function after ischemic injury [85]. Another signaling pathway that contributes to the fibrotic process is the AMP-activated protein kinase α (AMPKα) that phosphorylates β-catenin [86]. Two AMPKα isoforms have been studied: AMPKα1 and AMPKα2. AMPKα2 deletion has been associated to enhanced fibrosis and cardiac dysfunction [87] whereas AMPKα2 activation seems to limits fibrotic process related to pressure overload [88,89]. AMPKα1 modulates activities of cardiac fibroblast [90], and AMPKα loss in cardiac fibroblasts reduced fibrotic process related to pressure overload in mice [91]. GSK-3β is a serine/threonine kinase that is involved in the Wnt/β-catenin pathway. In fact, GSK-3β may contribute to β-catenin phosphorylation, triggering its degradation. In addition, GSK-3β may negatively interact with Smad-3, and GSK-3β deletion resulted in extensive fibrosis related to enhancement of TGF-β-Smad-3 signaling [92]. Indeed, several studies have highlighted the role of GSK-3β in the stabilization of Smad-3: their cytoplasmic physical interaction prevents Smad-3 nuclear translocation [93,94]. β-catenin cascade has been demonstrated to be involved in overload-pressure models and influences fibrotic process through fibroblast activity modulation; β-catenin deletion in fibroblasts was associated to reduced interstitial fibrosis, decreased ECM deposition and improved cardiac function [95]. Moreover, an important cross-talk between Wnt and TGF-β signaling is mediated by β-catenin and Smad-3 interaction: as a matter of fact, β-catenin nuclear translocation is also promoted by Smad-2 [96]; once into the nucleus, β-catenin combines with the Smad-2 and Smad-3 complex and with cyclic AMP-responsive element-binding protein (CBP) in order to regulate gene transcription, particularly a subset of genes stimulated by the TGF-β signaling [97]. In the non-canonical Wnt pathway, Wnt ligand binding results in the activation calcium/calmodulin-dependent kinase II (CamKII), protein kinase C (PKC), and calcineurin (CaN) or in the activation of Frizzled and Dishevelled receptors and the subsequent activation of Rho/ROCK and Rac/JNK [95]. Data on canonical Wnt/β-catenin suggest a role of this signal pathway in fibroblast proliferation and trans-differentiation following myocardial injury throughout the upregulation of pro-fibrotic genes [98,99]. In fact, a permanent activation of Wnt signaling in fibroblasts may promote myofibroblasts formation [69]. Both canonical and non-canonical Wnt/β-catenin pathways contribute and drive fibrosis: their activation correlates both with the onset and the severity of the diseases characterized by fibrosis. The development or the identification of possible inhibitors may represent an innovative and effective therapeutic approach for the management of fibrotic alterations.

## 4. Cardiovascular Imaging for the Evaluation of Myocardial Fibrosis

### 4.1. Introduction to Cardiac Magnetic Resonance 

In the last two decades, there have been huge hardware and software improvements allowing superior imaging resolution and increased definition of tissue characteristics in vivo. This allowed a wider implementation of advanced imaging techniques after MI, with both diagnostic and prognostic purposes. Above all, cardiac magnetic resonance (CMR) is the technique that provides a more precise evaluation of myocardial tissue characteristics, which justifies its increasing implementation in research and clinical practice. CMR allows non-invasive evaluation of myocardial volumes, myocardial function, and the physiopathology definition of heart disease in general [100,101,102]. CMR images depend on the relaxation time of the hydrogen nucleus protons subjected to a magnetic field. The longitudinal relaxation time (T1), and the transverse relaxation time (T2) correspond to the time required by protons to recover their longitudinal or transverse magnetization value at baseline. The intensity of the signal obtained from the individual tissues in the T1 and T2 sequences largely depends on the quantity of water present and on the pathological mechanisms that lead to an increase in its quantity (edema, fibrosis, infiltration). The use of the paramagnetic contrast medium Gadolinium induces a reduction in T1 times in the tissues, in relation to its concentration [103]. Gadolinium tends to accumulate in the fibrotic tissue, where it remains for a few minutes after its administration, and for this reason defined as Late Gadolinium Enhancement (LGE). 

### 4.2. Cardiac Magnetic Resonance Image Features

**Figure 3:**
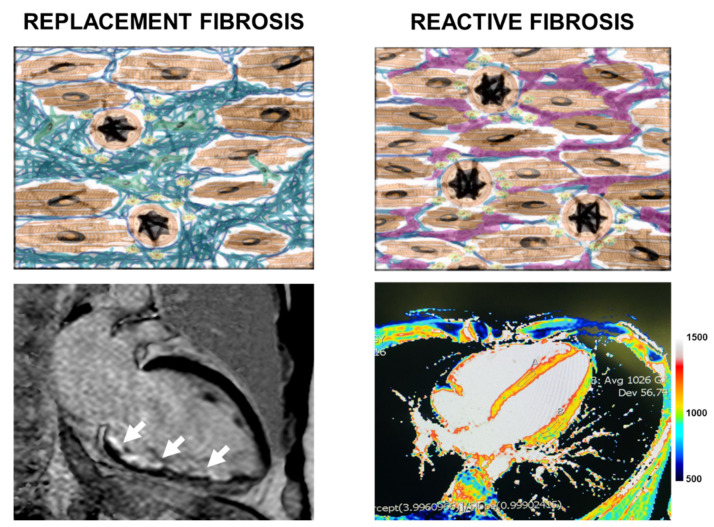
Description of the main patterns of myocardial fibrosis and the corresponding cardiac magnetic resonance images. Right panel: replacement fibrosis occurs in response to cardiomyocyte loss and can be observed in LGE image as a regional subendocardial scar (white arrows). Left panel: reactive fibrosis occurs without cardiomyocyte loss and can be observed in native T1 mapping image as raised native T1 values (1000–1200 ms).

In the heart, the fibrous areas will therefore appear hyperintense (i.e., white) in the T1 inversion recovery (IR) sequences, which are designed to attenuate the signal coming from the healthy myocardium and enhance the present LGE [104]. The type of distribution of the LGE according to specific patterns, identifies the physiopathological mechanism at the base of the fibrosis (i.e., ischemic or non-ischemic) [105,106]. In myocardial infarction the endo-epicardial distribution of the LGE correlates directly with the infarcted area in chronic phase [107]. CMR in patients with MI also allows identifying areas of noreflow and the presence of ventricular thrombi [108]. LGE identifies areas of fibrosis in a sensitive and reproducible way. However, LGE fails to give a quantitative definition of the fibrous areas and cannot identify the areas of reactive fibrosis due to its characteristic distribution [109]. In contrast, T1 mapping is a CMR method that guarantees a quantitative evaluation of the tissues analyzed [110]. In T1 mapping sequences, each pixel corresponds to the release time of a specific myocardial region, measured in milliseconds, giving a quantitative parameter of the analyzed myocardium. Before the administration of Gadolinium (native T1) the areas of diffuse fibrosis have a higher relaxation time compared to healthy tissue. After the administration of LGE the myocardial T1 is reduced, but to a lesser extent than the myocardial fibrosis region. Knowing the patient’s hematocrit and evaluating the T1 of the tissue under examination before and after administration of Gadolinium, we have the possibility of quantizing the myocardial ECV (extracellular volume). This value is increased in the myocardium, where fibrosis is more represented, and the cellular component decreased [111]. T1 mapping is able to identify early areas of myocardial fibrosis [112,113]. Furthermore, T1 mapping techniques are specific and sensitive in identifying areas of acute and chronic myocardial ischemia [114,115] (Figure 3).

## 5. Prognostic Impact of Fibrosis Identified by CMR

CMR has proven over the years to be a reliable tool in correlating image after MI with patient prognosis and adverse left ventricular remodeling. Di Bella et al., demonstrated in patient with previous MI that scar tissue extent, left ventricle dilatation, and wall motion abnormalities, together with age, are independently associated with cardiac death [116]. The severity of MI evaluated by a comprehensive CMR analysis (i.e., cine, T2-weighted, LGE imaging allowing assessment of ventricular function, area at risk, myocardial necrosis/fibrosis, microvascular obstruction [MVO], and myocardial hemorrhage) is correlated with adverse left ventricle remodeling and lack of functional recovery, in particular in hemorrhagic infarcts [117]. Assessment of MI severity by CMR in ST- segment elevation myocardial infarction patients is a strong prognostic marker, even if performed early as compared with CMR performed during a deferred follow-up [118]. Zhang et al., correlated T1 mapping and ECV after MI to adverse left ventricular remodeling. In particular, ECV is able to highlight areas of adverse remodeling even in the presence of MVO [119] Baritussio et al., identified a large prevalence of LGE in patients admitted after out-hospital cardiac arrest. In these patients, LGE correlated better with adverse events in follow-up than did LVEF and myocardial deformation [120]. Hence, independently from traditional imaging markers such as myocardial volume and bidimensional ventricular function, the prognostic role of myocardial fibrosis and its quantification has become more widely recognized and represents a new marker of poor prognosis in MI patients. 

## 6. Future Perspectives

### 6.1. Future Therapeutic Perspectives

Contemporary management of heart failure with loop diuretics, angiotensin-converting enzyme (ACE-I) inhibitors, angiotensin receptor blocker (ARB), beta-blockers, mineralocorticoid antagonists (MRA), ivabradine, hydralazine, isosorbide dinitrate, cardiac resynchronization therapy, and implantable cardioverter-defibrillator have been extensively tested and represent the standard of care according to international guidelines [121]. In addition, in recent years, multiple drugs focused on other pathways, such as the neuroendocrine system with sacubitril valsartan, metabolism with sodium glucose co-transporter 2 inhibitors, vascular function, and vasodilation with vericiguat, myocardial performance with omecamtiv mecarbil have been tested in clinical trials and are described in detail elsewhere [122]. Interestingly, some of these class of molecules have a direct effect on fibrotic process. Renin- angiotensin-aldosterone inhibitors (ACE-I, ARB, and MRA) have been demonstrated to reduce myocardial fibrosis development in humans, although with a scarce effect on fibrosis regression. [123,124,125,126,127,128,129,130]. Beta-blockers have been associated with reduction of cardiac fibrosis and mortality in mice model with an unknown underlying mechanism [131]. Ivabradine was associated with cardiomyocytes apoptosis reduction, fibrosis attenuation and reduction of circulating level of angiotensin II and aldosterone in animal models [132,133]. In addition, there are multiple future perspectives for acting directing on the myocardial fibrogenic process. Statins, likely for their inflammatory modulation effect, have been demonstrated to reduce cardiac fibrosis in animal models [134], but clinical evidence is actually discordant [135,136]. Peroxisome proliferator activated receptor (PPAR) agonists have an intense anti-inflammatory activity, and preclinical studies showed that they can reduce fibrosis and improve cardiac function [137]. TGF-b inhibitors as pirfenidone and tranilast, have been associated with cardiac fibrosis reduction in animal studies [138]. However, their clinical use is limited by safety concern related to their liver toxicity [139]. Matrix metalloproteinase (MMP) Inhibitors have been associated with cardiac fibrosis attenuation and left ventricle remodeling in animal models [140,141] but they failed to improve outcomes after MI in humans [142]. Relaxin is an endogenous hormone with pleiotropic effects including inhibition of TGF- b and Smad and modulation of MMPs. Serelaxin, the recombinant form of human relaxin-2 has shown anti-inflammatory and anti-fibrotic properties in different experimental models [143,144,145,146,147] but its beneficial effect has not emerged in clinical trials [148]. Although preclinical data on anti-fibrotic compounds are promising, their clinical application is limited and further studies are necessary. Engineered heart tissue derived from pluripotent stem cells represents a promising tool for cardiac repair although substantial doubts remain about its safety and optimal use. Hydrogel-based cell patch has been successfully evaluated in animal models [149,150] and in small human pilot studies with positive results [151]. Other non-cellular approaches to myocardial repair have been proposed. Alginate-hydrogel is a non-cellular inert compound that is directly injected into the myocardium to provide artificial scaffolding to the dilated left ventricle and modify its shape and size. This compound improved peak VO2, 6 min walking test and NYHA functional class in advanced chronic HF patients in a small randomized clinical trial [152].

### 6.2. Future Diagnostic Perspectives 

New technologies will further expand our ability to detect myocardial fibrosis and define its prognostic impact. Left ventricle function is governed by longitudinal and circumferential shortening, radial thickening, and rotational motion. The subendocardial fibers responsible for LV longitudinal contraction are the most vulnerable to wall stress and damage. The early decrease in LV longitudinal systolic function parallels or, in some cases, even precedes the development of diastolic abnormalities. This pattern of LV functional changes can be found, for example, in the preclinical stage of HFpEF. For this reason, the study of longitudinal deformation through re-elaboration of echocardiographic acquisitions has grown interest in recent times [153]. Global Longitudinal Strain (GLS) has been shown to predict major cardiovascular events better than LVEF, especially when the ejection fraction is mildly or moderately depressed [154]. GLS also proved to be an excellent predictor of major events in HFpEF [155]. CMR is the reference non-invasive imaging technique for evaluating fibrosis and myocardial remodeling. Diffusion tensor CMR (DT-CMR) is a novel technique that describes the three-dimensional heart microarchitecture, with the potential to assess the cardiomyocyte and sheetlet orientation [156]. Under normal conditions, the sheetlets align more wall-parallel in diastole and more wall-perpendicular in systole. In remodeling hearts, the sheetlets have altered systolic deformation and reduced mobility [157]. Four-dimensional flow CMR is an emerging technology used to visualize and quantify intra-cardiac blood flow. This new technology can better describe flow route and myocardium energetics that might be impaired in subclinical LV dysfunction, representing early non-invasive markers of dysfunction and myocardial damage [158].

## 7. Conclusions

After myocardial infarction, the activation of multiple cellular and molecular pathways resulting in fibrosis represents a physiological response to ischemic injury and necrosis. Both an exaggerated fibrotic response, which is associated with increased wall stiffness and risk of heart failure, or a reduced fibrotic response, which, in turn, is associated with ventricular dilation and risk of wall rupture, may impair the healing process after a myocardial infarction. Advanced imaging techniques such as cardiac magnetic resonance allow superior characterization of myocardial tissue and fibrosis quantification in vivo, providing valuable diagnostic and prognostic information after myocardial infarction.

## Data Availability

The data presented in this study are available in manuscript.
